# Grb7, a Critical Mediator of EGFR/ErbB Signaling, in Cancer Development and as a Potential Therapeutic Target

**DOI:** 10.3390/cells8050435

**Published:** 2019-05-10

**Authors:** Pei-Yu Chu, Yu-Ling Tai, Tang-Long Shen

**Affiliations:** 1 Department of Plant Pathology and Microbiology, National Taiwan University, Taipei 10617, Taiwan; areteka@gmail.com (P.-Y.C.); d98633002@ntu.edu.tw (Y.-L.T.); 2 Department of Urology, University of Texas Southwestern Medical Center, Dallas, TX 75390, USA; 3 Center for Biotechnology, National Taiwan University, Taipei 10617, Taiwan

**Keywords:** growth factor receptor bound protein-7, ERBB family, epidermal growth factor receptor, cancer development, prognostic marker, therapeutic target

## Abstract

The partner of activated epidermal growth factor receptor (EGFR), growth factor receptor bound protein-7 (Grb7), a functionally multidomain adaptor protein, has been demonstrated to be a pivotal regulator for varied physiological and pathological processes by interacting with phospho-tyrosine-related signaling molecules to affect the transmission through a number of signaling pathways. In particular, critical roles of Grb7 in erythroblastic leukemia viral oncogene homolog (ERBB) family-mediated cancer development and malignancy have been intensively evaluated. The overexpression of Grb7 or the coamplification/cooverexpression of Grb7 and members of the ERBB family play essential roles in advanced human cancers and are associated with decreased survival and recurrence of cancers, emphasizing Grb7′s value as a prognostic marker and a therapeutic target. Peptide inhibitors of Grb7 are being tested in preclinical trials for their possible therapeutic effects. Here, we review the molecular, functional, and clinical aspects of Grb7 in ERBB family-mediated cancer development and malignancy with the aim to reveal alternative and effective therapeutic strategies.

## 1. Introduction

Through studies of binding targets of tyrosine phosphorylated EGFR, growth factor receptor bound protein 7 (Grb7), a 535-amino acid multidomain adaptor protein, was initially identified as an EGFR binding partner [1]. Of note, the 535-amino acid protein refers to mouse Grb7, which was originally identified from a mouse library, while the human *GRB7* gene encodes a 532-amino acid protein [1,2]. Functionally, Grb7 has been indicated to be involved in the regulation of multiple signal transduction cascades during various physiological and pathological processes [3]. Structurally, Grb7 and its related family members Grb10 and Grb14 exhibit conserved domains, such as an N-terminal proline-rich motif, a central GM (named for Grb and Mig) region, and a C-terminal Src-homology 2 (SH2) domain, allowing for multiple protein–protein and protein–lipid interactions [3,4]. Through interactions with the ERBB protein family, Grb7 has been found to transmit and amplify the oncogenic ERBB protein family-mediated signal transduction cascades that play key roles in regulating cancer development [5,6,7].

Additionally, the human *GRB7* gene is located on chromosome 17q12, in close proximity to the cancer-associated *ERBB2* gene, which has been identified as an *ERBB2* amplicon [8], suggesting the coamplification and coexpression of Grb7 and ERBB2 contribute to cancer development. The copy number and expression levels of both *GRB7* and *ERBB2* are highly increased in many human cancers [9,10]. Moreover, an increased protein expression level of Grb7 is highly correlated with *ERBB2* gene amplification and ERBB2 oncoprotein expression in invasive cancers [11].

**Figure 1:**
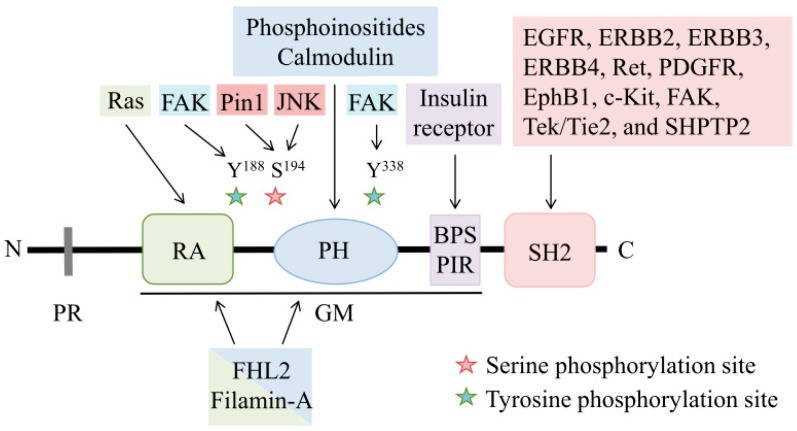
Schematic structure of Grb7. Grb7 contains an N-terminal proline-rich (PR) region, a GM (Grb and Mig) region, and a C-terminal SH2 domain. The N-terminal PR region contains five minimal consensus proline-rich (PxxP) motifs that are responsible for SH3-mediated protein–protein interactions. The GM region is composed of an RA (Ral GDS/AF6 or Ras-Associating) domain, a pleckstrin homology (PH) domain, a BPS (Between the PH and SH2 domains)/PIR (phosphotyrosine interacting region) that binds to Ras, FHL2, Filamin-A, phosphoinositides, calmodulin, or insulin receptors et al. In the GM region, there are two major tyrosine phosphorylation sites at Y188 and the Y338, which can be phosphorylated by focal adhesion kinase (FAK). One serine phosphorylation site at S194 is phosphorylated by c-Jun N-terminal kinase (JNK). The phosphotyrosine motif of numerous molecules, such as the epidermal growth factor receptor (EGFR) family or FAK, has been indicated in their association with the C-terminal SH2 domain of Grb7.

**Figure 2:**
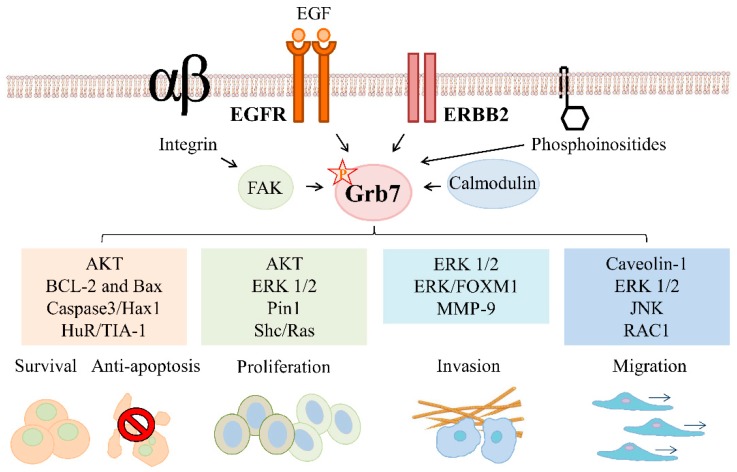
Signal transduction and function of Grb7 in ERBB family-mediated cancer development. ERBB family-mediated Grb7 signal transduction modulates survival, anti-apoptosis, proliferation, migration, and invasion of cancer. For instance, epidermal growth factor (EGF) stimulated-Grb7-Hax1 interactions regulate cancer apoptosis. EGF-induced Grb7 tyrosine phosphorylation activates Ras-GTPases and extracellular signal-regulated kinases 1/2 (ERK1/2) contributes to cancer proliferation. The ERBB2/Grb7-regulated Shc/Ras signal modulates cancer proliferation. The EGF signal mediates cell migration by an enhanced association and recruitment of Grb7 and Caveolin-1. Grb7 modulates cancer invasion by mediating EGF/EGFR signal-mediated ERK activation or matrix metallopeptidase 9 (MMP-9) expression. Grb7 is also involved in the integrin/FAK-, phosphoinositide-, or calmodulin-mediated cell functions. By interacting with FAK or phosphoinositides, Grb7 regulates cell migration. In addition, the interaction between Grb7 and calmodulin modulates cell angiogenic activity.

This review will provide an overview of the basic properties and regulatory mechanisms of Grb7, as summarized in Figure 1, and it will discuss the role of Grb7 signals in ERBB family-mediated cancer development, as illustrated in Figure 2. Furthermore, a summary of the potential clinical applications of Grb7 as a prognostic marker and a therapeutic target in ERBB family-driven cancers is also provided.

### 1.1. Structure of Grb7

#### 1.1.1. Proline-Rich Region

The N-terminus of the Grb7 protein contains a proline-rich region with five minimal consensus PxxP motifs for Src homology region 3 (SH3)-mediated protein–protein interactions [4]. However, the binding proteins of the proline-rich region of Grb7 remain unclear. Although the proline-rich region of the Grb7 adaptor protein family is highly conserved, the proline-rich region of Grb10 has been shown to interact with the SH3 domain of Abelson tyrosine kinase (c-Abl), whereas the SH3 domains of Grb2, Fyn (a Src family tyrosine kinase), and the p85 regulatory subunit of phosphoinositide 3 kinase (PI3-Kinase) do not [12].

#### 1.1.2. GM Region

The GM region possesses approximately 300 amino acids with 50% amino acid identity between the Grb7 adaptor protein family and Mig-10 of *Caenorhabditis elegans* [13]. Functionally, Mig-10 regulates cell migration during embryonic development in *Caenorhabditis elegans*, suggesting an important role of Grb7 in cell migration [13,14]. There are three domains within the GM region, including an RA (Ral GDS/AF6 or Ras-Associating) domain, a pleckstrin homology (PH) domain, a BPS (between PH and SH2 domains), and a PIR (phosphotyrosine interacting region) [13,15].

Sequence analysis identifies the structural conservation of the RA domain in the Grb7 adaptor protein family [15], suggesting the potential ability of Grb7 to interact with small GTPases of the Ras superfamily and in regulating Ras-mediated cancer progression. Of note, using glutathione S-transferase (GST)-tagged Ras binding domain (RBD) of the c-Raf kinase protein (GST-Raf-RBD) pull-down analysis, our study was the first to identify a positive relationship between Grb7 and active Ras in response to EGF stimulation [5]. Moreover, EGF-induced Grb7-mediated Ras activation and ERK phosphorylation are required for breast cancer development [5]. In addition, both the RA domain and PH domain of Grb7 have been reported to act together in the regulation of cell functions [16,17]. For instance, the RA-PH domain of Grb7 interacts with the four and a half LIM domains isoform 2 (FHL2), a cancer-associated transcription regulator [16]. The interaction between the RA-PH domain of Grb7 and Filamin-A, an actin-binding protein, has been indicated to be involved in forming membrane ruffles in response to EGF stimulation, suggesting a critical role of Grb7-mediated signals in cytoskeletal remodeling [17].

The PH domain has been shown to govern cellular signaling events, such as vesicle trafficking or cytoskeletal organization [18], and the PH domain of Grb7 has been demonstrated to have protein and/or lipid binding abilities and regulate various cellular functions [19,20]. Indeed, our study indicated that the PH domain of Grb7 exhibits a high affinity for specific phosphatidylinositol phosphates, especially the D3- and D5-phosphoinositides, in a focal adhesion kinase (FAK)/PI3-kinase signaling axis-dependent manner [19]. The interaction between the PH domain of Grb7 and phosphoinositides displays a significant effect on integrin-mediated cell migration [19]. By analyzing protein–protein interactions, deletion of an amphiphilic basic amino acid sequence that is located in the proximal region of the PH domain of Grb7 ablates the calmodulin-binding ability of Grb7 [20]. Functionally, the interaction between Grb7 and calmodulin enables cell angiogenic activity, implying the functional effect of Grb7-mediated signals on neovascularization [20].

The BPS/PIR region of Grb7 has been shown to interact with the activated insulin receptor in the SH2 domain of Grb7 dependent and independent interactions [21], implying a potential role of Grb7 in insulin receptor-mediated signals. Regarding the similarity of the BPS/PIR region across the Grb7 adaptor protein family, the BPS/PIR region of both Grb10 [22] and Grb14 [23] has been shown to interact with the insulin receptor. Indeed, the BPS/PIR region of Grb10 and Grb14 has been indicated to be involved in the modulation of insulin- and/or insulin-like growth factor 1-mediated signals [24,25].

#### 1.1.3. SH2 Domain

For the spatial and temporal effects of Grb7 on the regulation of multiple signal transduction cascades, the SH2 domain of Grb7 functions in a critical role for Grb7 by binding to activated receptor tyrosine kinases/nonreceptor tyrosine kinases or other tyrosine-phosphorylated signaling proteins [3]. The SH2 domain of Grb7 has been demonstrated to bind to various phosphotyrosine motifs of its binding partners, such as EGFR [1], ERBB2 [26], ERBB3 [27], ERBB4 [27], Ret [28], platelet-derived growth factor receptor (PDGFR) [29], EphB1 [30], c-Kit [31], FAK [32], Tek/Tie2 [33], SHPTP2 [34], and so on. Moreover, the interaction between the SH2 domain of Grb7 and the phosphotyrosine motif of signaling proteins results in distinct cellular outcomes or tumorigenic functions. For instance, synthetic Grb7-binding peptides, which target to the SH2 domain of Grb7, inhibit the interaction between the Grb7 and ERBB family in breast cancer [7] and FAK activation in pancreatic cancer [6]. However, a seemingly contradictory dual role of Grb7-mediated cellular functions has emerged in several studies. In invasive esophageal carcinoma, the wild-type Grb7 protein and its variant Grb7v isoform that lacks an SH2 domain in the C terminus have been found to exhibit different phosphorylation statuses in response to EGF stimulation [35]. Clearly, both Grb7-mediated and Grb7v-mediated cell proliferation occur in an ERK signal-dependent manner, whereas the wild-type Grb7 regulates cell migration via a JNK-dependent signal [36]. Functionally, Grb7v exhibits a higher oncogenic effect than wild-type Grb7 on the anchorage-independent growth of ovarian cancer [36]. On the other hand, Grb7, but not Grb7v, facilitates the migration and invasion of ovarian cancer [36]. Displaying different phosphorylation statuses and regulating distinct downstream targets of Grb7 and Grb7v might cause different effects of Grb7 and Grb7v on cancer functions. In different types and stages of cancers, the structural statuses and the phosphorylation statuses of Grb7 might also lead to a seemingly contradictory dual role of the SH2 domain of Grb7 in Grb7-mediated oncogenesis. Together, these studies emphasize the critical role of the SH2 domain of Grb7 on physiological and pathological processes.

### 1.2. Regulatory Mechanisms of Grb7

#### 1.2.1. Phosphorylation

Grb7 can be tyrosine phosphorylated by various kinases, including EGFR [37], EphB1 [30], Tek/Tie2 [33], FAK [19,38,39], and so on. In human esophageal carcinoma, the tyrosine phosphorylation of Grb7 has been detected in response to EGF or fibronectin stimulation [37]. Functionally, the SH2 dominant-negative fragment of Grb7 ablates the fibronectin-mediated tyrosine phosphorylation of Grb7, resulting in a reduction of cancer migration [37]. Consistently, EphB1 receptor tyrosine kinase binds to Grb7 and phosphorylates the tyrosine residue of Grb7, which is strongly correlated with EphB1-mediated cell migration [30]. Likewise, the SH2 dominant-negative fragment of Grb7 attenuates the regulatory effect of EphB1 on cell migration [30].

Similarly, a specific tyrosine residue (Tyr-1100) of Tek receptor tyrosine kinase functions as a functional docking site for binding to Grb7, whereas the mutated Tyr-1100 of Tek abolishes the Grb7-binding ability and tyrosine phosphorylation of Grb7 by Tek [33]. On the other hand, tyrosine phosphorylation of Grb7 can also be detected when Grb7 binds to HS-1-associated protein X-1 (HAX-1), a cytoskeletal-associated protein that is overexpressed in metastatic cancers [40], suggesting the potential role of tyrosine phosphorylation of Grb7 in cancer development.

In addition to the functional effects of receptor tyrosine kinases on Grb7, nonreceptor tyrosine kinase FAK also endows a critical impact on the tyrosine phosphorylation of Grb7. FAK is a critical upstream factor in Grb7-mediated signaling pathways that enable activation by growth factor receptors or integrins during cancer development [41]. The binding of Grb7 to the autophosphorylation site, Tyr-397, of FAK results in integrin-mediated cell migration, whereas overexpression of the SH2 domain of Grb7 ablates cell migration towards fibronectin [32]. Mechanistically, the SH2 domain of Grb7 has been shown to affect Grb7-mediated cell migration by regulating Grb7 targeting to focal contacts [38]. Interestingly, substitution of the SH2 domain of Grb7 with a focal adhesion targeting sequence from FAK restores Grb7-mediated cell migration [38]. Additionally, FAK kinase activity but not the Src family kinases (non-receptor tyrosine kinases) are capable of tyrosine phosphorylation of Grb7 that subsequently leads to FAK-mediated cell migration [19,38]. Consistently, our studies indicated that FAK enables phosphorylation of Grb7 on at least two tyrosine residues, Tyr-188 and Tyr-338, upon formation of a complex with FAK [39]. The FAK/Grb7 complex regulates the phosphorylation of paxillin and ERK1/2 [39]. Functionally, the formation of the FAK/Grb7 complex or FAK-mediated Grb7 phosphorylation are required for cancer malignancy, such as cell migration, proliferation, and anchorage-independent growth [39]. In contrast, tyrosine phosphorylation-deficient mutants of Grb7 ablate integrin-dependent cell migration and proliferation [39]. These studies indicate a critical role of FAK in the regulation of the phosphorylation of Grb7 as well as Grb7-mediated cancer development.

Serine/threonine phosphorylation has also been reported to be involved in Grb7-mediated cellular functions. In response to EGF [8] or heregulin [27] stimulation, the presence of serine and threonine phosphorylation of Grb7 has been investigated in several cellular biological studies. Interestingly, our recent study provided a negative regulatory mechanism for Grb7 via JNK-mediated phosphorylation of Grb7, in which the phospho-Ser194-Pro motif of Grb7 required for binding to peptidyl-prolyl cis/trans isomerase Pin1 facilitates the proteasome-mediated degradation of Grb7 protein [42]. Functionally, Pin1-mediated Grb7 protein stability regulates cell cycle progression at the G2-M phase [42]. These studies illustrate the varied regulatory mechanisms of Grb7 protein phosphorylation, which take part in physiological and pathophysiological processes.

#### 1.2.2. Localization

In response to upstream signal activation and the recruitment of downstream signaling molecules, the subcellular localization of Grb7 has been investigated in the cytoplasm, focal adhesions, stress granules, and the nucleus [19,38,43,44]. Consistent with the functions of Grb7 and Grb7v as adaptor proteins, the subcellular localization of both Grb7 and Grb7v is primarily in the cytoplasm [36]. Furthermore, deletion of the SH2 domain of Grb7 ablates the subcellular localization of Grb7 in focal contacts, thereby resulting in inhibition of Grb7-mediated cell migration [38]. Although the PH domain of Grb7 is not necessary for the recruitment of Grb7 to focal contacts, the interaction between the PH domain of Grb7 and phospholipids may recruit Grb7 to the plasma membrane [19]. The other close link between its subcellular localization and the functional effects for Grb7 is the interaction of Grb7 and Hu antigen R that is found in the stress granules, indicating Grb7 is an integral component of stress granules [43]. Additionally, Grb7 has been indicated as a key mediator in the formation of the nuclear-cytoplasmic export complex in an EGF signal-dependent manner [45]. In response to EGF stimulation, nuclear SHP-2-mediated dephosphorylated nuclear Grb7 recruits KOR mRNA and Hu antigen R, subsequently leading to the nuclear export of KOR mRNA [45]. Intriguingly, the calmodulin-binding domain of Grb7, which is located in the proximal region of the PH domain, exhibits a specific sequence with high similarity to nuclear localization signals and regulates the nuclear localization of Grb7 [44]. Consistently, the deletion of the calmodulin-binding domain of Grb7 ablates its nuclear localization [44]. In contrast, calmodulin antagonist treatment contributes to the enhancement of Grb7 in the nucleus [44], suggesting that the nuclear localization of Grb7 is modulated by calmodulin.

#### 1.2.3. Dimerization

The dimerization or well-defined oligomerization of proteins is a common characteristic of signaling proteins. Although most SH2 domains exist mainly as a monomer, several studies have suggested that the SH2 domain of Grb7 can exist as a homodimer [46,47,48]. Indeed, the dimeric status of the SH2 domain of Grb7 has been demonstrated by using sedimentation equilibrium ultracentrifugation [46]. By size-exclusion chromatography, a single point mutation of Grb7 converting phenylalanine 511 to an arginine, F511R, results in a monomeric status of the SH2 domain of Grb7 [46]. Functionally, a dimerization-defective mutant F511R of Grb7 fails to regulate Ras activity and ERK1/2 phosphorylation [5], suggesting a critical functional effect of the structural status of Grb7 on Grb7-mediated signaling and function. Actually, it has been indicated that the dimerization of the SH2 domain of Grb7 affects the structural integrity and the phosphorylated tyrosine peptide ligand-binding ability of Grb7 [48]. Furthermore, a tyrosine phosphorylation-mimic mutation of Grb7 exhibited a reduced thermal stability and a dimerization deficiency [48], suggesting that the tyrosine phosphorylation status of Grb7 might affect the dimerization of Grb7. By analyzing the crystal structure of the SH2 domain of Grb7, it was found that the effect on Grb7 dimeric status of a synthetic Grb7-binding peptide, which targets the SH2 domain of Grb7, provides a potential strategy for treating Grb7-mediated cancer progression [47].

## 2. Grb7 Signaling in ERBB Family-Mediated Cancer Development

### 2.1. Grb7 Signaling in ERBB Family-Mediated Cancer Survival and Proliferation

#### 2.1.1. Grb7 Signaling in ERBB Family-Mediated Cancer Survival

Grb7- and ERBB family/Grb7-mediated signal transduction cascades have been reported to facilitate cancer cell survival through the regulation of mitochondria-associated apoptotic pathways [49]. In contrast, knockdown of *GRB7* expression decreases the expression of the anti-apoptotic B-cell lymphoma-2 (BCL-2) and increases the expression of the pro-apoptotic BCL2-associated X, leading to a significant increase in apoptosis [49,50]. Additionally, Grb7 functions as a translational regulator in the regulation of stress granule formation, which is a process used to reduce stress-induced damage and allows the cell to survive [43]. Upon experiencing stresses, the recruitment of hypo-phosphorylated Grb7 by RNA-binding protein Hu antigen R (HuR) to stress granules has been shown to stabilize TIA-1, a cytotoxic granule associated RNA binding protein, that aggregates with and enhances the association of HuR with TIA-1, leading to the aggregation of and the enhancement of stress granule integrity [43]. When the cell stress is terminated, activated FAK-mediated hyperphosphorylated Grb7 leaves HuR and other stress granule components, leading to the disruption of stress granule integrity [43]. Additionally, FAK-mediated Grb7 phosphorylation is involved in the regulation of cell survival in a nutrient-deficient condition [39], indicating Grb7 is a key regulator of cell survival. These studies suggest that the distinct phosphorylation statuses of Grb7, which are regulated by FAK, play different roles in response to the dynamic changes of cells. Moreover, Grb7 has been shown to facilitate the binding of protein tyrosine kinase Syk to stress granules [51]. Subsequently, Syk enhances the clearance of stress granules through the formation of autophagosomes [51], suggesting the critical effects of Grb7 on the recruitment of specific proteins to stress granules to regulate cell survival.

Moreover, several ERBB family-mediated signals contribute to Grb7-mediated cell survival as well as anti-apoptosis in cancer cells. For instance, EGF signaling stimulates an interaction in mitochondria between Grb7 and HS1 associated protein X1 (Hax1) isoform 1, an anti-apoptotic protein, and subsequently regulates cancer apoptosis by affecting caspase3-mediated Hax1 cleavage [52]. Moreover, the RNA interference (RNAi)-mediated gene silencing of *Grb7* together with lapatinib, a dual tyrosine kinase inhibitor that blocks EGFR and ERBB2 signals, contributes to the inhibition of protein kinase B (PKB, also known as AKT) activity and decreased cell viability [53]. Moreover, cosilencing of ERBB2 co-amplified genes, including ERBB2 and GRB7, results in increased apoptosis in cancers [53].

#### 2.1.2. Grb7 Signaling in ERBB Family-Mediated Cancer Proliferation

Upregulation of oncogenic ERBB family-mediated signals stimulates cancer proliferation. The expression of Grb7, one of the most important mediators in ERBB family-mediated signaling, is significantly correlated with cell cycle progression and anchorage-independent growth [5,39,42,54], highlighting Grb7 as a critical mediator in ERBB family-mediated cancer proliferation. Indeed, short hairpin RNA-mediated knockdown of *GRB7* expression reduces cancer proliferation and anchorage-independent growth in ERBB2/Grb7-overexpressing breast cancers [5,55]. Moreover, the ERBB2/Grb7-mediated Shc/Ras signal transduction cascade has been reported to regulate the proliferation of ERBB2-overexpressing breast cancers [5,54].

On the other hand, knockdown of *GRB7* expression has been indicated to attenuate ERBB2 and AKT phosphorylation in breast cancer cells, affecting ERBB2-mediated cancer growth in vivo [56]. Notably, targeted knockdown of genes in the *ERBB2* amplicon, including *GRB7*, leads to an additive effect on the decreased cell-cycle progression and cancer proliferation [57], emphasizing the importance of *GRB7* coamplified with *ERBB2* in their contribution to cancer proliferation.

The protein stability, protein binding ability, and phosphorylation of Grb7 are also critical for Grb7- or ERBB family/Grb7- mediated cancer proliferation. Indeed, Grb7 protein stability modulated by the peptidyl-prolyl cis/trans isomerase Pin1 has been implicated in the regulation of cell cycle progression at the G2-M phase [42]. Additionally, the specific Grb7 peptide targeting the SH2 domain of Grb7 efficiently reduces the formation of the Grb7 and ERBB family complex [7] and leads to the attenuation of 3D culture colony formation, which reflects a combination of anti-proliferative and/or pro-apoptotic effects on cancer cells [50]. Furthermore, Grb7 peptides targeting the SH2 domain of Grb7 impaired the phosphorylation of ERK 1/2 in EGF-stimulated cancers [50]. Reportedly, GRB7v, a variant of Grb7 that displays a substitution of the SH2 domain with a hydrophobic sequence, has also been shown to greatly increase cancer proliferation as well as anchorage-independent growth by regulation of ERK signaling [36]. Of note, tyrosine phosphorylation-deficient mutants of Grb7 have been shown to regulate the phosphorylation of AKT, ERK1/2, and paxillin, and subsequently ablate cancer proliferation and anchorage-independent growth [39]. In addition, EGF-triggered Grb7 tyrosine phosphorylation activates Ras-GTPases and promotes the phosphorylation of ERK1/2, which, in turn, stimulates cancer proliferation and growth [5]. These studies indicate critical effects of Grb7- and ERBB family/Grb7-mediated signal transduction cascades on cell fate determination by regulating cell cycle progression, anchorage-independent growth, and proliferation.

### 2.2. Grb7 Signaling in ERBB Family-Mediated Cancer Migration, Invasion, and Metastasis

#### 2.2.1. Grb7 Signaling in ERBB Family-Mediated Cancer Migration

Grb7- or ERBB family/Grb7- mediated signal transduction cascades have been implicated in the promotion of cancer migration. Indeed, it has been shown that high Grb7 expressing cancer cells exhibit a higher migratory ability than low Grb7 expressing cells [36,39,58]. Moreover, tyrosine phosphorylation of Grb7 has also been shown to regulate cancer migration [39]. In these cancer cells, mitogen-activated protein kinases (MAPKs), such as JNK or ERK, are crucial downstream signals regulated by Grb7 in promoting cancer movement [36,39,59]. Consistently, knockdown or inhibition of Grb7 expression or the specific kinase activity of Grb7-mediated downstream signals abrogates Grb7-mediated cancer migration [36,39,59]. Additionally, several upstream signals contribute to Grb7-mediated cancer migration. For instance, EGF triggers the tyrosine phosphorylation of Caveolin-1, which, in turn, recruits Grb7 to Caveolin-1 and facilitates cell migration [60]. As expected, short hairpin RNA-mediated knockdown of *GRB7* expression reduces gap closure migration of cancers in response to the EGF treatment [55]. However, no significant difference in cell migration between Grb7 knockdown cells and control cells in the absence of EGF treatment suggests that EGF signaling is critical in Grb7-stimulated cell migration [55]. Additionally, the Grb7 peptide that ablates the interaction between Grb7 and protein tyrosine kinases, including the ERBB family, significantly attenuates cancer movement [6]. In ERBB2 positive cancer cells, knockdown of Grb7 has been found to decrease integrin-mediated RAC1 activation as well as integrin-mediated cell movement [54]. Beyond this, Grb7 regulates cell migration by interactions with various receptor tyrosine kinases, non-receptor tyrosine kinases, and phospholipids. For instance, the association of Grb7 with EphB1 [30] or FAK [32] enhances cell migration, whereas overexpression of the Grb7 SH2 domain reduces EphB1- or FAK-mediated cell migration [30,32]. Intrinsically, the deletion of the Grb7 SH2 domain, which ablates the distribution of Grb7 in focal contacts, might affect the Grb7-stimulated cell migration [38]. Additionally, the PH domain of Grb7 has also been shown to regulate cell migration by binding to phosphoinositides, especially D3- and D5-phosphoinositides [19].

#### 2.2.2. Grb7 Signaling in ERBB Family-Mediated Cancer Invasion and Metastasis

The coexpression of Grb7 with the ERBB family, especially EGFR and ERBB2, is significantly correlated with the invasion of advanced esophageal carcinomas [61]. Moreover, the overexpression of Grb7 is positively correlated with the presence of lymph node metastases of esophageal carcinomas [37], suggesting a critical role of Grb7 in metastatic progression. Due to the identification of metastasis-acquired ERBB2 alterations in patients with breast cancer brain metastases, Grb7 has been recently indicated as one of the most recurrently upregulated genes in cancers [62]. These studies highlight Grb7 as a critical mediator in ERBB family-mediated cancer invasion and metastasis. In addition, specific Grb7 peptides targeting the SH2 domain of Grb7, which blocks EGF/EGFR signal-mediated ERK activation [50], or knockdown of *GRB7*, which ablates MMP-9 expression [49], attenuate cancer invasion. Moreover, GRB7-mediated ERK/forkhead box M1 (FOXM1) signaling has been suggested to be an oncogenic convergence in the stimulation of cancer invasion [59]. Consistently, inhibition of the specific kinase activity of Grb7-mediated downstream signals significantly reduces Grb7-mediated cancer invasion [59]. Moreover, an interaction between Grb7 and calmodulin has been indicated in the regulation of cell angiogenic activity, suggesting a critical role of Grb7 in cancer metastatic spread and the development of neovascularization [20]. In a mouse model, Grb7 peptide inhibitor, which selectively blocks the interactions between Grb7 and ERBB family proteins or FAK [6,7] as well as blocking the phosphorylation of Grb7 protein, significantly ablates the peritoneal metastasis of pancreatic cancer cells [6]. These studies highlight the important functional effects of Grb7 and the ERBB family/Grb7 on cancer malignancy by modulating cancer invasive and metastatic abilities.

## 3. Cooverexpression and Coamplification of the Grb7 and ERBB Family in Clinical Applications

Cooverexpression or coamplification of Grb7 and the ERBB family has been clinically investigated in human breast cancers [62,63], cervical cancers [49], invasive Barrett’s carcinoma [64], advanced esophageal carcinoma [61], and gastric cancers [10,65]. Numerous empirical studies have verified that ErbB family/Grb7-regulated signaling contributes to the malignant characteristics of cancers, emphasizing Grb7 as a promising prognostic marker and a potential therapeutic target in ERBB family-mediated cancer. Indeed, both *GRB7* and *ERBB2* are key genes that predict the recurrence of breast cancer in clinical trials [66,67]. Based on the critical role of Grb7 in cancer, a Grb7 peptide targeting the specific Grb7 SH2 phosphotyrosine binding site that prevents Grb7-mediated signaling appears to be a promising targeted therapeutic candidate against malignant cancers. Furthermore, combination treatment with the Grb7 peptide and ERBB family-targeted drugs display cooperative functions in cancer therapy. The following will describe and discuss in detail the development of a Grb7 expression-based prognostic assay and Grb7-related therapeutics in ERBB family-mediated cancers.

### 3.1. Grb7 and Coamplification/Cooverexpression of Grb7 and the ERBB Family in Clinical Studies

Clinical studies have indicated that upregulation of Grb7 is significantly correlated with the recurrence and low survival of patients with cancer [50,63,68,69], suggesting critical roles of Grb7 during cancer development and treatment. Moreover, several studies have revealed that the consistent upregulation of Grb7 and the ERBB family facilitates the development of cancer malignancies. By cloning receptor targets using the tyrosine phosphorylated C-terminus of EGFR as a probe, Grb7 was initially identified as an EGFR binding protein [1]. Actually, the coexpression of Grb7 with EGFR was significantly related to advanced esophageal carcinomas with extramucosal invasion, whereas this phenomenon was not induced by the sole expression of Grb7 or EGFR [61]. Intrinsically, our studies indicated that EGF-induced de novo Grb7 tyrosine phosphorylation/activation is essential for the tumorigenicity [5], suggesting that expression and activation of both Grb7 and EGFR might collaboratively function as a critical point in cancer developmental processes.

By using comparative genomic hybridization microarray analyses of human chromosome 17q12 in the *ERBB2* amplicon, where *GRB7* is located on the chromosome next to *ERBB2*, the copy number and expression levels of genes within the *ERBB2* amplicon, such as *GRB7* and *ERBB2*, were highly increased in human breast cancers [9], gastric cancers [10], high grade intraepithelial neoplasia, and invasive Barrett’s carcinoma [64]. Moreover, the elevated protein expression of Grb7 is strongly correlated with *ERBB2* gene amplification as well as ERBB2 overexpression in invasive breast cancer [11]. Cooverexpression of Grb7 and ERBB2 are strongly associated with a worse prognosis than that of ERBB2 overexpression alone [63], suggesting the clinical significance of both Grb7 and ERBB2 genes/proteins expression in cancer development. Several empirical studies showed that Grb7 associates with ERBB2 [54] and facilitates ERBB2-mediated cancer migration and proliferation [54,70]. Of note, our study indicates that the anti-cancer effect is synergized by cotreatment with Herceptin, an ERBB2-targeted monoclonal antibody, plus Grb7 knockdown in ERBB2^+^ breast cancers [5], reflecting the tight correlation between Grb7 and ERBB2 in cancer progression and treatment.

### 3.2. Grb7 as a Prognostic Marker in Cancer

In clinical prognosis, metastasis is one of major risk factors affecting mortality in patients with diagnosed malignant cancers. In agreement with the clinical relevance of the Grb7 expression level in cancer malignancy, overexpression of Grb7 was significantly correlated with the lymph node-positive status of cancer metastasis [37]. Additionally, the elevated expression of Grb7 was highly correlated with late clinical stage or low survival in patients with cancers, including breast cancers [63,68] and esophageal carcinoma [37]. In addition, several studies indicated that the inhibition of Grb7 expression reduced motility/invasion and promoted the apoptosis-mediated cell death of malignant cancers [5,39,50]. Additionally, our studies and others have further investigated the EGF/EGFR-mediated tyrosine phosphorylation of Grb7 that is involved in aggressive and malignant features of cancers [5,37,39]. Clinically, high expression of both Grb7 and the ERBB family, especially ERBB2, are highly associated with a poor prognosis of patients with breast cancer [63]. These studies highlighted Grb7 as a valuable prognostic marker of survival in ERBB family-regulated cancers.

Predictive factors of cancer drug response and recurrence are also important indicators during cancer treatment. In triple-negative breast cancer (TNBC) patients treated with standard adjuvant anthracycline and taxane therapy, the five-year recurrence rates were 10.5% and 20.4% in the low and the high *GRB7* RNA expression groups, respectively [69]. In addition, high *GBR7* expression was correlated with resistance to neoadjuvant doxorubicin and taxane therapy [69]. The expression levels of both Grb7 and ERBB2 provide valuable and predictive information based on an empirical relationship between Grb7 and the ERBB family in malignant cancer development and treatment. In clinical trials, both *GRB7* and *ERBB2* have been indicated as two key genes in the 21 gene recurrence score assay to predict the recurrence of tamoxifen-treated breast cancer [66,67,71]. Together, these studies indicate high *GRB7* expression is a highly significant predictor for recurrence after cancer treatment.

### 3.3. Grb7 as a Therapeutic Target in Cancer

In agreement with the importance of the SH2 phosphotyrosine binding site of Grb7 in Grb7-mediated signal transduction cascades and cancer malignancy, the specific Grb7 peptide targeting the SH2 phosphotyrosine binding site of Grb7 efficiently blocks Grb7-mediated signal transduction cascades. Using phage display random peptide libraries as ligands to the SH2 domain of Grb7, Pero et al. found that peptides containing a nonphosphorylated Tyr-X-Asn (YXN) motif exhibited high specificity and binding affinity to the SH2 domain of Grb7 [7]. Subsequently, the Grb7-peptide 18-no arms thioether (termed G7-18NATE) was developed and displayed high binding specificity for the SH2 domain of Grb7, but not the SH2 domain of the closely related Grb2 or Grb14 [7]. By protein structure analysis, a specific amino acid arginine 462 (R642) in Grb7 has been identified as mediating the interaction between the SH2 domain of Grb7 and G7-18NATE [72]. In contrast, the Grb7 R462S mutant exhibits a significant decrease in binding affinity for G7-18NATE [72]. G7-18NATE has been found to ablate the interaction between Grb7 and the ERBB family in a dose-dependent manner in breast cancer cells [7]. Of note, G7-18NATE also selectively blocked the interaction between Grb7 and FAK and blocked the tyrosine phosphorylation of Grb7, as well as significantly reducing the migration and peritoneal metastasis of pancreatic cancer in preclinical studies [6]. Additionally, the finding of no significant deleterious effects of G7-18NATE on nonmalignant cells [73] or animals [6] suggests the long-term safety and well tolerability of peptides targeting Grb7 in patients with cancers. Based on the crystal structure analysis, several studies improved the affinity of specific peptide inhibitors of Grb7, such as G7-B1 and G7-B4, over G7-18NATE [74,75]. Indeed, a new series of bicyclic G7 peptides exhibited an enhanced binding affinity to the SH2 domain of Grb7 [74,75]. According to the structure-based progression, a new optimized bicyclic peptide that displays high specificity for the SH2 domain of Grb7 has been recently developed to block the interaction between Grb7 and the ERBB family in cancer cells [76]. By improvement of the drug delivery system, the conjugation of G7-18NATE to cell-penetrating peptides, such as Penetratin, has exhibited effective cytosolic delivery of G7-18NATE and migration inhibition of breast cancer cells [77,78]. Due to Grb7 being a potential therapeutic target in cancer, these studies improved the efficiency of the Grb7 inhibitor and promoted the development of Grb7-targeted therapies.

Understanding the detailed regulatory mechanisms of Grb7 in cancer development can provide alternative methods for cancer therapy. Recently, a study indicated a negative correlation between miR-193a-3p and *GRB7* in ovarian cancers [79]. miR-193a-3p has been shown to directly target the 3′ UTR of *GRB7* [79], suggesting possible Grb7-targeted therapies by tumor suppressor miRNAs. Targeting Grb7-mediated signaling has also been indicated in cancer therapy. The tyrosine phosphorylation of EGFR was ablated in *GRB7* knockdown ERBB2^+^ breast cancer, suggesting the effects of Grb7-mediated EGFR activation on the malignancy of ERBB2^+^ breast cancer [80]. Taking advantage of this novel regulatory phenomenon, panitumumab, a humanized monoclonal antibody targeting EGFR, has been shown to significantly decrease the proliferation of ERBB2^+^ breast cancer [80].

As numerous studies have illustrated, Grb7 amplifies oncogenic signals to promote cancer development, thereby highlighting that targeting both Grb7 and Grb7-amplified oncogenic signals provides a synergistic therapeutic effect on anti-cancer therapy. Clinically, patients with ERBB2^+^ breast cancer treated with two approved therapies targeting ERRB2, Herceptin and lapatinib, tend to experience relapses or malignant outcomes [81], indicating that improvements in anti-cancer treatment are urgently required. Due to the upregulation of Grb7, which is correlated with recurrence and low survival in patients with cancer [50,63,68,69], combination treatments targeting both Grb7 and oncogenic signals provide potential therapeutic strategies. Actually, our study indicates that there are synergistic anti-cancer effects of cotreatment with Herceptin and shRNA targeting *GRB7* in ERRB2^+^ breast cancers [5]. Additionally, combination treatment with Grb7 peptide and Herceptin or doxorubicin display cooperative anti-cancer effects [73]. Together, these studies suggest that combination treatment with shRNA or peptides targeting Grb7 and chemotherapy, or targeted therapy improves the efficiency of anti-cancer therapy.

## 4. Conclusions

Grb7 as a functionally multidomain adaptor is essential for cancer development by binding to phospho-tyrosine-related oncogenic signaling molecules to amplify oncogenic signal transduction cascades. In particular, the multifunctional features of Grb7 have been emphasized as being involved in ERBB family-mediated cancer malignancy. However, the functional role and pathological significance of Grb7 in vivo, during the entire process of ERBB family-mediated cancer development, as well as tumor microenvironment formation need to be illustrated. Based on preclinical and clinical studies, the upregulation of Grb7 in numerous human cancers highlights Grb7 as a potential prognostic marker and a therapeutic target. Currently, the use of the expression of both *GRB7* and *ERBB* in the prediction of the recurrence of breast cancer has been validated in a clinical trial. Additionally, shRNA or peptides targeting Grb7 expression or the SH2 phosphotyrosine binding site display promising anti-cancer effects in preclinical studies. Since the Grb7 protein interacts with several oncogenic protein tyrosine kinases, especially the ERBB family, combination therapy with peptides targeting Grb7 and protein tyrosine kinase-targeting agents/drugs can be viewed as an innovative therapeutic intervention. Of note, the detailed regulatory mechanisms, side effects, proper dose, tolerability, as well as treatment-induced resistant cancers against molecules targeting Grb7 or in combination with protein tyrosine kinase-targeting agents/drugs remains to be evaluated. Collectively, the molecular mechanisms and cellular functions of Grb7 in ERBB family-mediated cancer have provided valuable basic and clinical information for targeting Grb7 or Grb7-amplified oncogenic signals to improve the outcomes of human cancer cases.

## References

[1] Margolis B., Silvennoinen O., Comoglio F., Roonprapunt C., Skolnik E., Ullrich A., Schlessinger J. (1992). High-efficiency expression/cloning of epidermal growth factor-receptor-binding proteins with Src homology 2 domains. Proc. Natl. Acad. Sci. USA.

[2] Lucas-Fernandez E., Garcia-Palmero I., Villalobo A. (2008). Genomic organization and control of the grb7 gene family. Curr. Genomics.

[3] Shen T.L., Guan J.L. (2004). Grb7 in intracellular signaling and its role in cell regulation. Front. Biosci..

[4] Han D.C., Shen T.L., Guan J.L. (2001). The Grb7 family proteins: Structure, interactions with other signaling molecules and potential cellular functions. Oncogene.

[5] Chu P.Y., Li T.K., Ding S.T., Lai I.R., Shen T.L. (2010). EGF-induced Grb7 recruits and promotes Ras activity essential for the tumorigenicity of Sk-Br3 breast cancer cells. J. Biol. Chem..

[6] Tanaka S., Pero S.C., Taguchi K., Shimada M., Mori M., Krag D.N., Arii S. (2006). Specific peptide ligand for Grb7 signal transduction protein and pancreatic cancer metastasis. J. Natl. Cancer Inst..

[7] Pero S.C., Oligino L., Daly R.J., Soden A.L., Liu C., Roller P.P., Li P., Krag D.N. (2002). Identification of novel non-phosphorylated ligands, which bind selectively to the SH2 domain of Grb7. J. Biol. Chem..

[8] Stein D., Wu J., Fuqua S.A., Roonprapunt C., Yajnik V., D’Eustachio P., Moskow J.J., Buchberg A.M., Osborne C.K., Margolis B. (1994). The SH2 domain protein GRB-7 is co-amplified, overexpressed and in a tight complex with HER2 in breast cancer. EMBO J..

[9] Kauraniemi P., Barlund M., Monni O., Kallioniemi A. (2001). New amplified and highly expressed genes discovered in the ERBB2 amplicon in breast cancer by cDNA microarrays. Cancer Res..

[10] Varis A., Wolf M., Monni O., Vakkari M.L., Kokkola A., Moskaluk C., Frierson H., Powell S.M., Knuutila S., Kallioniemi A. (2002). Targets of gene amplification and overexpression at 17q in gastric cancer. Cancer Res..

[11] Bivin W.W., Yergiyev O., Bunker M.L., Silverman J.F., Krishnamurti U. (2017). GRB7 Expression and Correlation with HER2 Amplification in Invasive Breast Carcinoma. Appl. Immunohistochem. Mol. Morphol..

[12] Frantz J.D., Giorgetti-Peraldi S., Ottinger E.A., Shoelson S.E. (1997). Human GRB-IRbeta/GRB10. Splice variants of an insulin and growth factor receptor-binding protein with PH and SH2 domains. J. Biol. Chem..

[13] Manser J., Roonprapunt C., Margolis B.C. (1997). elegans cell migration gene mig-10 shares similarities with a family of SH2 domain proteins and acts cell nonautonomously in excretory canal development. Dev. Biol..

[14] Manser J., Wood W.B. (1990). Mutations Affecting Embryonic-Cell Migrations in Caenorhabditis-Elegans. Dev. Genet..

[15] Wojcik J., Girault J.A., Labesse G., Chomilier J., Mornon J.P., Callebaut I. (1999). Sequence analysis identifies a ras-associating (RA)-like domain in the N-termini of band 4.1/JEF domains and in the Grb7/10/14 adapter family. Biochem. Biophys. Res. Commun..

[16] Siamakpour-Reihani S., Argiros H.J., Wilmeth L.J., Haas L.L., Peterson T.A., Johnson D.L., Shuster C.B., Lyons B.A. (2009). The cell migration protein Grb7 associates with transcriptional regulator FHL2 in a Grb7 phosphorylation-dependent manner. J. Mol. Recognit..

[17] Paudyal P., Shrestha S., Madanayake T., Shuster C.B., Rohrschneider L.R., Rowland A., Lyons B.A. (2013). Grb7 and Filamin-a associate and are colocalized to cell membrane ruffles upon EGF stimulation. J. Mol. Recognit..

[18] Lemmon M.A., Ferguson K.M., Abrams C.S. (2002). Pleckstrin homology domains and the cytoskeleton. FEBS Lett..

[19] Shen T.L., Han D.C., Guan J.L. (2002). Association of Grb7 with phosphoinositides and its role in the regulation of cell migration. J. Biol. Chem..

[20] Li H., Sanchez-Torres J., del Carpio A.F., Nogales-Gonzalez A., Molina-Ortiz P., Moreno M.J., Torok K., Villalobo A. (2005). The adaptor Grb7 is a novel calmodulin-binding protein: Functional implications of the interaction of calmodulin with Grb7. Oncogene.

[21] Kasus-Jacobi A., Bereziat V., Perdereau D., Girard J., Burnol A.F. (2000). Evidence for an interaction between the insulin receptor and Grb7. A role for two of its binding domains, PIR and SH2. Oncogene.

[22] He W., Rose D.W., Olefsky J.M., Gustafson T.A. (1998). Grb10 interacts differentially with the insulin receptor, insulin-like growth factor I receptor, and epidermal growth factor receptor via the Grb10 Src homology 2 (SH2) domain and a second novel domain located between the pleckstrin homology and SH2 domains. J. Biol. Chem..

[23] Kasus-Jacobi A., Perdereau D., Auzan C., Clauser E., Van Obberghen E., Mauvais-Jarvis F., Girard J., Burnol A.F. (1998). Identification of the rat adapter Grb14 as an inhibitor of insulin actions. J. Biol. Chem..

[24] Stein E.G., Gustafson T.A., Hubbard S.R. (2001). The BPS domain of Grb10 inhibits the catalytic activity of the insulin and IGF1 receptors. FEBS Lett..

[25] Depetris R.S., Hu J., Gimpelevich I., Holt L.J., Daly R.J., Hubbard S.R. (2005). Structural basis for inhibition of the insulin receptor by the adaptor protein Grb14. Mol. Cell.

[26] Janes P.W., Lackmann M., Church W.B., Sanderson G.M., Sutherland R.L., Daly R.J. (1997). Structural determinants of the interaction between the erbB2 receptor and the Src homology 2 domain of Grb7. J. Biol. Chem..

[27] Fiddes R.J., Campbell D.H., Janes P.W., Sivertsen S.P., Sasaki H., Wallasch C., Daly R.J. (1998). Analysis of Grb7 recruitment by heregulin-activated erbB receptors reveals a novel target selectivity for erbB3. J. Biol. Chem..

[28] Pandey A., Liu X., Dixon J.E., Di Fiore P.P., Dixit V.M. (1996). Direct association between the Ret receptor tyrosine kinase and the Src homology 2-containing adapter protein Grb7. J. Biol. Chem..

[29] Yokote K., Margolis B., Heldin C.H., Claesson-Welsh L. (1996). Grb7 is a downstream signaling component of platelet-derived growth factor alpha- and beta-receptors. J. Biol. Chem..

[30] Han D.C., Shen T.L., Miao H., Wang B., Guan J.L. (2002). EphB1 associates with Grb7 and regulates cell migration. J. Biol. Chem..

[31] Thommes K., Lennartsson J., Carlberg M., Ronnstrand L. (1999). Identification of Tyr-703 and Tyr-936 as the primary association sites for Grb2 and Grb7 in the c-Kit/stem cell factor receptor. Biochem. J..

[32] Han D.C., Guan J.L. (1999). Association of focal adhesion kinase with Grb7 and its role in cell migration. J. Biol. Chem..

[33] Jones N., Master Z., Jones J., Bouchard D., Gunji Y., Sasaki H., Daly R., Alitalo K., Dumont D.J. (1999). Identification of Tek/Tie2 binding partners. Binding to a multifunctional docking site mediates cell survival and migration. J. Biol. Chem..

[34] Keegan K., Cooper J.A. (1996). Use of the two-hybrid system to detect the association of the protein-tyrosine-phosphatase, SHPTP2, with another SH2-containing protein, Grb7. Oncogene.

[35] Tanaka S., Mori M., Akiyoshi T., Tanaka Y., Mafune K., Wands J.R., Sugimachi K. (1998). A novel variant of human Grb7 is associated with invasive esophageal carcinoma. J. Clin. Invest.

[36] Wang Y., Chan D.W., Liu V.W., Chiu P., Ngan H.Y. (2010). Differential functions of growth factor receptor-bound protein 7 (GRB7) and its variant GRB7v in ovarian carcinogenesis. Clin. Cancer Res..

[37] Tanaka S., Sugimachi K., Kawaguchi H., Saeki H., Ohno S., Wands J.R. (2000). Grb7 signal transduction protein mediates metastatic progression of esophageal carcinoma. J. Cell Physiol..

[38] Han D.C., Shen T.L., Guan J.L. (2000). Role of Grb7 targeting to focal contacts and its phosphorylation by focal adhesion kinase in regulation of cell migration. J. Biol. Chem..

[39] Chu P.Y., Huang L.Y., Hsu C.H., Liang C.C., Guan J.L., Hung T.H., Shen T.L. (2009). Tyrosine phosphorylation of growth factor receptor-bound protein-7 by focal adhesion kinase in the regulation of cell migration, proliferation, and tumorigenesis. J. Biol. Chem..

[40] Siamakpour-Reihani S., Peterson T.A., Bradford A.M., Argiros H.J., Haas L.L., Lor S.N., Haulsee Z.M., Spuches A.M., Johnson D.L., Rohrschneider L.R. (2011). Grb7 binds to Hax-1 and undergoes an intramolecular domain association that offers a model for Grb7 regulation. J. Mol. Recognit..

[41] Tai Y.L., Chen L.C., Shen T.L. (2015). Emerging roles of focal adhesion kinase in cancer. BioMed Res. Int..

[42] Tai Y.L., Tung L.H., Lin Y.C., Lu P.J., Chu P.Y., Wang M.Y., Huang W.P., Chen K.C., Lee H., Shen T.L. (2016). Grb7 Protein Stability Modulated by Pin1 in Association with Cell Cycle Progression. PLoS ONE.

[43] Tsai N.P., Ho P.C., Wei L.N. (2008). Regulation of stress granule dynamics by Grb7 and FAK signalling pathway. EMBO J..

[44] Garcia-Palmero I., Villalobo A. (2012). Calmodulin regulates the translocation of Grb7 into the nucleus. FEBS Lett..

[45] Tsai N.P., Lin Y.L., Tsui Y.C., Wei L.N. (2010). Dual action of epidermal growth factor: Extracellular signal-stimulated nuclear-cytoplasmic export and coordinated translation of selected messenger RNA. J. Cell Biol..

[46] Porter C.J., Wilce M.C., Mackay J.P., Leedman P., Wilce J.A. (2005). Grb7-SH2 domain dimerisation is affected by a single point mutation. Eur. Biophys. J..

[47] Porter C.J., Matthews J.M., Mackay J.P., Pursglove S.E., Schmidberger J.W., Leedman P.J., Pero S.C., Krag D.N., Wilce M.C., Wilce J.A. (2007). Grb7 SH2 domain structure and interactions with a cyclic peptide inhibitor of cancer cell migration and proliferation. BMC Struct. Biol..

[48] Peterson T.A., Benallie R.L., Bradford A.M., Pias S.C., Yazzie J., Lor S.N., Haulsee Z.M., Park C.K., Johnson D.L., Rohrschneider L.R. (2012). Dimerization in the Grb7 protein. J. Mol. Recognit..

[49] Zhao H.B., Zhang X.F., Jia X.L., Wang H.B. (2017). Grb7 is over-expressed in cervical cancer and facilitate invasion and inhibit apoptosis in cervical cancer cells. Pathol. Res. Pract..

[50] Giricz O., Calvo V., Pero S.C., Krag D.N., Sparano J.A., Kenny P.A. (2012). GRB7 is required for triple-negative breast cancer cell invasion and survival. Breast Cancer Res. Treat..

[51] Krisenko M.O., Higgins R.L., Ghosh S., Zhou Q., Trybula J.S., Wang W.H., Geahlen R.L. (2015). Syk Is Recruited to Stress Granules and Promotes Their Clearance through Autophagy. J. Biol. Chem..

[52] Qian L., Bradford A.M., Cooke P.H., Lyons B.A. (2016). Grb7 and Hax1 may colocalize partially to mitochondria in EGF-treated SKBR3 cells and their interaction can affect Caspase3 cleavage of Hax1. J. Mol. Recognit..

[53] Sahlberg K.K., Hongisto V., Edgren H., Makela R., Hellstrom K., Due E.U., Moen Vollan H.K., Sahlberg N., Wolf M., Borresen-Dale A.L. (2013). The HER2 amplicon includes several genes required for the growth and survival of HER2 positive breast cancer cells. Mol. Oncol..

[54] Pradip D., Bouzyk M., Dey N., Leyland-Jones B. (2013). Dissecting GRB7-mediated signals for proliferation and migration in HER2 overexpressing breast tumor cells: GTP-ase rules. Am. J. Cancer Res..

[55] Lim R.C., Price J.T., Wilce J.A. (2014). Context-dependent role of Grb7 in HER2+ve and triple-negative breast cancer cell lines. Breast Cancer Res. Treat..

[56] Bai T., Luoh S.W. (2008). GRB-7 facilitates HER-2/Neu-mediated signal transduction and tumor formation. Carcinogenesis.

[57] Kao J., Pollack J.R. (2006). RNA interference-based functional dissection of the 17q12 amplicon in breast cancer reveals contribution of coamplified genes. Genes Chromosomes Cancer.

[58] Haran M., Chebatco S., Flaishon L., Lantner F., Harpaz N., Valinsky L., Berrebi A., Shachar I. (2004). Grb7 expression and cellular migration in chronic lymphocytic leukemia: A comparative study of early and advanced stage disease. Leukemia.

[59] Chan D.W., Hui W.W., Cai P.C., Liu M.X., Yung M.M., Mak C.S., Leung T.H., Chan K.K., Ngan H.Y. (2012). Targeting GRB7/ERK/FOXM1 signaling pathway impairs aggressiveness of ovarian cancer cells. PLoS ONE.

[60] Lee H., Volonte D., Galbiati F., Iyengar P., Lublin D.M., Bregman D.B., Wilson M.T., Campos-Gonzalez R., Bouzahzah B., Pestell R.G. (2000). Constitutive and growth factor-regulated phosphorylation of caveolin-1 occurs at the same site (Tyr-14) in vivo: Identification of a c-Src/Cav-1/Grb7 signaling cassette. Mol. Endocrinol..

[61] Tanaka S., Mori M., Akiyoshi T., Tanaka Y., Mafune K., Wands J.R., Sugimachi K. (1997). Coexpression of Grb7 with epidermal growth factor receptor or Her2/erbB2 in human advanced esophageal carcinoma. Cancer Res..

[62] Priedigkeit N., Hartmaier R.J., Chen Y., Vareslija D., Basudan A., Watters R.J., Thomas R., Leone J.P., Lucas P.C., Bhargava R. (2017). Intrinsic Subtype Switching and Acquired ERBB2/HER2 Amplifications and Mutations in Breast Cancer Brain Metastases. JAMA Oncol..

[63] Nadler Y., Gonzalez A.M., Camp R.L., Rimm D.L., Kluger H.M., Kluger Y. (2010). Growth factor receptor-bound protein-7 (Grb7) as a prognostic marker and therapeutic target in breast cancer. Ann. Oncol..

[64] Walch A., Specht K., Braselmann H., Stein H., Siewert J.R., Hopt U., Hofler H., Werner M. (2004). Coamplification and coexpression of GRB7 and ERBB2 is found in high grade intraepithelial neoplasia and in invasive Barrett’s carcinoma. Int. J. Cancer.

[65] Kwon M.J., Kim R.N., Song K., Jeon S., Jeong H.M., Kim J.S., Han J., Hong S., Oh E., Choi J.S. (2017). Genes co-amplified with ERBB2 or MET as novel potential cancer-promoting genes in gastric cancer. Oncotarget.

[66] Paik S., Shak S., Tang G., Kim C., Baker J., Cronin M., Baehner F.L., Walker M.G., Watson D., Park T. (2004). A multigene assay to predict recurrence of tamoxifen-treated, node-negative breast cancer. N. Engl. J. Med..

[67] Sparano J.A., Gray R.J., Makower D.F., Pritchard K.I., Albain K.S., Hayes D.F., Geyer C.E., Dees E.C., Perez E.A., Olson J.A. (2015). Prospective Validation of a 21-Gene Expression Assay in Breast Cancer. N. Engl. J. Med..

[68] Ramsey B., Bai T., Hanlon Newell A., Troxell M., Park B., Olson S., Keenan E., Luoh S.W. (2011). GRB7 protein over-expression and clinical outcome in breast cancer. Breast Cancer Res. Treat..

[69] Sparano J.A., Goldstein L.J., Childs B.H., Shak S., Brassard D., Badve S., Baehner F.L., Bugarini R., Rowley S., Perez E.A. (2011). Relationship between quantitative GRB7 RNA expression and recurrence after adjuvant anthracycline chemotherapy in triple-negative breast cancer. Clin. Cancer Res..

[70] Saito M., Kato Y., Ito E., Fujimoto J., Ishikawa K., Doi A., Kumazawa K., Matsui A., Takebe S., Ishida T. (2012). Expression screening of 17q12-21 amplicon reveals GRB7 as an ERBB2-dependent oncogene. FEBS Lett..

[71] Paik S. (2007). Development and clinical utility of a 21-gene recurrence score prognostic assay in patients with early breast cancer treated with tamoxifen. Oncologist.

[72] Watson G.M., Lucas W.A.H., Gunzburg M.J., Wilce J.A. (2017). Insight into the Selectivity of the G7-18NATE Inhibitor Peptide for the Grb7-SH2 Domain Target. Front. Mol. Biosci..

[73] Pero S.C., Shukla G.S., Cookson M.M., Flemer S., Krag D.N. (2007). Combination treatment with Grb7 peptide and Doxorubicin or Trastuzumab (Herceptin) results in cooperative cell growth inhibition in breast cancer cells. Br. J. Cancer.

[74] Gunzburg M.J., Ambaye N.D., Del Borgo M.P., Perlmutter P., Wilce J.A. (2013). Design and Testing of Bicyclic Inhibitors of Grb7-Are Two Cycles Better Than One?. Biopolymers.

[75] Gunzburg M.J., Kulkarni K., Watson G.M., Ambaye N.D., Del Borgo M.P., Brandt R., Pero S.C., Perlmutter P., Wilce M.C., Wilce J.A. (2016). Unexpected involvement of staple leads to redesign of selective bicyclic peptide inhibitor of Grb7. Sci. Rep..

[76] Watson G.M., Kulkarni K., Sang J., Ma X., Gunzburg M.J., Perlmutter P., Wilce M.C.J., Wilce J.A. (2017). Discovery, Development, and Cellular Delivery of Potent and Selective Bicyclic Peptide Inhibitors of Grb7 Cancer Target. J. Med. Chem..

[77] Watson G.M., Kulkarni K., Brandt R., Del Borgo M.P., Aguilar M.I., Wilce J.A. (2017). Shortened Penetratin Cell-Penetrating Peptide Is Insufficient for Cytosolic Delivery of a Grb7 Targeting Peptide. ACS Omega.

[78] Ambaye N.D., Lim R.C.C., Clayton D.J., Gunzburg M.J., Price J.T., Pero S.C., Krag D.N., Wilce M.C.J., Aguilar M.I., Perlmutter P. (2011). Uptake of a Cell Permeable G7-18NATE Construct Into Cells and Binding With the Grb7-SH2 Domain. Biopolymers.

[79] Chen K., Liu M.X., Mak C.S., Yung M.M., Leung T.H., Xu D., Ngu S.F., Chan K.K., Yang H., Ngan H.Y. (2018). Methylation-associated silencing of miR-193a-3p promotes ovarian cancer aggressiveness by targeting GRB7 and MAPK/ERK pathways. Theranostics.

[80] Luoh S.W., Wagoner W., Wang X., Hu Z., Lai X., Chin K., Sears R., Ramsey E. (2019). GRB7 dependent proliferation of basal-like, HER-2 positive human breast cancer cell lines is mediated in part by HER-1 signaling. Mol. Carcinog..

[81] Rexer B.N., Arteaga C.L. (2012). Intrinsic and acquired resistance to HER2-targeted therapies in HER2 gene-amplified breast cancer: Mechanisms and clinical implications. Crit. Rev. Oncog..

